# Results from a randomized controlled trial investigating effectiveness of a community-based intervention on empowerment of people with severe mental illness

**DOI:** 10.1007/s00127-025-02879-3

**Published:** 2025-04-01

**Authors:** Annabel Sandra Mueller-Stierlin, Thomas Becker, Nils Greve, Anke Hänsel, Katrin Herder, Anne Kohlmann, Jutta Lehle, Uta Majewsky, Friedrich Meixner, Elke Prestin, Melanie Pouwels, Nadja Puschner, Sabrina Reuter, Mara Schumacher, Stefanie Wöhler, Reinhold Kilian

**Affiliations:** 1 Institut für Epidemiologie und Medizinische Biometrie, Universität Ulm, Schwabstraße 13, 89075 Ulm, Germany; 2 Klinik für Psychiatrie und Psychotherapie II, Universität Ulm, Lindenallee 2, 89312 Günzburg, Germany; 3 Seniorprofessur Klinik und Poliklinik für Psychiatrie und Psychotherapie, Universität Leipzig, Semmelweisstraße 10, 04103 Leipzig, Germany; 4 Dachverband Gemeindepsychiatrie e.V, Richartzstraße 12, 50667 Köln, Germany; 5 Macromedia University of Applied Sciences, Naststraße 11, 70376 Stuttgart, Germany

**Keywords:** Assertive community treatment, Severe mental illness, Empowerment, Psychosocial interventions, Multiprofessional teams

## Abstract

**Purpose:**

The effectiveness of community mental health services with respect to enhancing empowerment among patients with severe mental illness (SMI) has rarely been investigated. In this multicenter trial the effectiveness of a community mental health intervention (acronym: GBV) added to treatment as usual (TAU) compared to TAU alone was investigated.

**Methods:**

In a randomized controlled multicenter trial with twelve sites spread across Germany, people living with SMI aged 18–82 years were investigated over 24 months. The trial was conducted from 2020 to 2023, a time period affected by the Covid-19 pandemic. The intervention was delivered by multiprofessional GBV teams based on the Functional Assertive Community Treatment (FACT) program and was supplemented by strategies that increase the degree of self-determination. The primary outcome was measured by the Assessment of Empowerment in Patients with Affective and Schizophrenic Disorders (EPAS). Difference in difference (DiD) effect sizes were estimated on an intention-to-treat basis.

**Results:**

A total of 929 persons with SMI were randomly assigned to the GBV plus TAU intervention (*n* = 470) or to TAU alone (*n* = 459). The dropout rate over 24 months amounted to 28%. DiD effect sizes over 24 months indicate significant treatment effects for empowerment (d = 0.27; 95% CI = 0.14 0.40). Serious adverse events (SAE) were reported for 15 (3.2%) participants in the GBV + TAU vs. 17 (3.7%) in the TAU group.

**Conclusion:**

The addition of GBV to TAU, for patients with SMI, can be recommended as an effective measure to improve key psychosocial outcomes in mental health care settings across Germany.

**Trial registration:**

German Clinical Trial Register, DRKS00019086. Registered on 3 January 2020, https://drks.de/search/de/trial/DRKS00019086.

## Introduction

Beyond the burden of psychopathological symptoms and functional impairment many people with severe mental illness (SMI) suffer from a lack of mastery and control over their lives [1–3]. Feelings of helplessness, among people with SMI, apart from being sequels of psychopathological impairment may also arise from adverse interaction with mental health and social care systems [4, 5]. Therefore, the empowerment of SMI patients with a view to increasing their capacity to manage and control their lives has become a key target in modern mental health care concepts [6–9]. The theory of empowerment [10–12] suggests that people, under deprived living conditions, may develop feelings of learned helplessness with regard to their capacity to manage their own lives and that this process can be reversed by interventions that strengthen self-efficacy expectations and increase trust in the capability to deal with the demands of daily life. This process is commonly defined as the personal dimension of“empowerment” [10]. Since the empowerment process depends on patients’ experience of being able to manage daily life demands, interventions for supporting empowerment need to be provided in their living environments. Community mental health services (CMHS) provided by multi-professional teams are widely regarded as the most suitable organizational setting for empowerment interventions for people with SMI [13]. However, in spite of good evidence of the effectiveness and safety of CHMS regarding the reduction of hospital admissions and the improvement of social functioning [14–16], the effectiveness of CMHS with respect to enhancing empowerment among patients with SMI has rarely been investigated [17].

In the current study, we aimed to investigate the effectiveness of a community mental health care intervention called “Gemeindepsychiatrische Basisversorgung” (GBV) on the improvement of empowerment in people with SMI with a more appropriate study design [18]. The intervention is based on the Functional Assertive Community Treatment (FACT) [19] program developed and implemented in the Netherlands and several Scandinavian countries [20–22], supplemented by strategies and measures that increase the degree of autonomy and self-determination in the lives of people and enable them to represent their interests independently and self-responsibly. Multiprofessional GBV teams focused their work on the enhancement of empowerment and recovery and organized network meetings according to the concept of Open Dialogue [23]. As primary outcome we hypothesized that perceived empowerment on the personal dimension in participants receiving the GBV intervention in addition to treatment as usual (TAU), over a period of 24 months, would improve more than in study participants receiving TAU alone comprising rather fragmented and difficult to navigate services.

## Methods

### Study framework and funding

The investigation was funded as an independent evaluative study of the GBV intervention by the Innovation Fund of the Federal Joint Committee (G-BA) under the funding code 01NVF18028.

### Study design

We conducted an un-blinded randomized controlled trial including participants at twelve sites across Germany. Study duration was 24 months with five points of assessment at six-month intervals. The first participant was included in June 2020, and the last participant finished the trial in May 2023. Details of the study design and methods are outlined in the published study protocol [18].

This study was approved by the Ethics Committee of Ulm University on August 28, 2019 (application number: 259/19) and by local ethics committees. This trial was registered at the German Clinical Trial Register DRKS00019086.

### Intervention

Participants in the intervention group received a community based mental health care program “Gemeindepsychiatrische Basisversorgung” (GBV) provided by a multi-professional mental health care team in addition to treatment as usual (TAU) [18]. The GBV program is based on the functional assertive community treatment (FACT) model developed in the Netherlands [19], and it combines long-term individual case management focused on the enhancement of the participants’ self-help capabilities (empowerment) using multidisciplinary service planning, assertive outreach and a 24 h 7 days-a-week crisis service [18].

The GBV teams aimed at supporting service users, using the concepts of empowerment and recovery, in identifying pathways to successfully shape their individual lives, improve their quality of living and increase their opportunities of social participation. For this purpose, the case-manager was expected to support the service user in dealing with everyday life issues (e.g. organization of everyday life, social contacts, partnership, job related problems, economic problems). We expect that this support helps the service user to develop control experiences which are the basis of the empowerment process. This was achieved through a foundational attitude of mutual appreciation, an orientation, among study participants, towards both individual preferences and social resources and a joint search for potential solutions to problems using dialogue-based communication. This implied a communication style based on equal terms and a flow of process management controlled as far as possible by service users. If possible, all stakeholders (service users, service providers, informal carers/ relatives, using a ‘trialogue-based approach’) were involved, and their respective concepts were accepted respecting controversy and integrated as far as possible. Informed by the Open Dialogue approach, dialogical conversation was promoted in network meetings with service users, their relatives and carers as well as professionals. GBV services were offered in a need-adapted approach and financed on a fee for service basis. The focus and the intensity of the intervention varies between service users and over time depending on the service users’ current mental health state and preferences.

### Treatment as usual (TAU)

Study participants had full access to general and psychiatric health and psychosocial care provided by the German health and social care systems and covered by the statutory health insurance, the pension funds and the social benefit system. This comprises somatic and psychiatric inpatient care including emergency services provided by general or psychiatric hospitals, general and psychiatric outpatient treatment provided by office-based family doctors and specialized physicians (including psychiatrists), ambulatory psychotherapy provided by office-based psychologists, and community mental health services provided by psychiatric outpatient clinics. In addition, participants had access to occupational rehabilitation and supported housing if they were eligible according to the rules of the social code.

In spite of a high level of resources spent on mental health care the implementation of integrated CMHS in Germany is underdeveloped when compared with the UK, the Netherlands and Scandinavian countries [24] and thus, treatment as usual comprises rather fragmented and difficult to navigate services. Among other reasons, the German social code regulations were considered by many experts to constitute a barrier against the implementation of integrated care: While in Germany medical psychiatric services are mainly financed by statutory or private health insurances, vocational services are financed by unemployment funds or pension funds and psychosocial and residential services are financed by individual social benefits or taxes. Though, in order to reduce these barriers, a reformation of the social security code part 5 §§ 140a in 2004 allowed provision of integrated medical and psychosocial services by community mental health providers which previously were only allowed to provide psychosocial services [17].

### Sample and recruitment process

Persons were eligible for study participation if they were members of one of the statutory health insurance funds that belonged to the study consortium, if they were 18 years or older, if they had an ICD-10 diagnosis of psychotic disorder (F20 – F29), affective disorder (F30 – F39), anxiety disorder (F40 – F48), behavioral disorder (F50 – F 59), or personality disorder (F60 – F69) and if they fulfilled the criteria of a severe mental illness which were multi-dimensional defined on the basis of a previous study [18] to identify participants with high psychosocial impairment (Health of the Nations Outcome Scale (HoNOS) [25] total sum score ≥ 12) and several unmet needs (Camberwell Assessment of Need (CAN) [26], unmet need score ≥ 4), and who potentially might benefit from integrated care services regarding the primary outcome (Assessment of Empowerment in Patients with Affective and Schizophrenic Disorders (EPAS) score ≤ 3,3 [18], by Exclusion criteria were a primary diagnosis of an organic mental disorder or an addiction disorder.

Persons potentially eligible for study participation (caseness for a prior diagnosis of a mental disorder as outlined above) were contacted either by their health insurance or by mental health care providers and invited to take part in the screening process to assess eligibility. Enrolment of participants, including the written informed consent procedure and the subsequent eligibility screening, was performed by local GBV service providers between May 7, 2020 (first participant) and May 19, 2021 (last participant).

### Impact of the Covid-19 pandemic

The provision of psychiatric services in general and the implementation of the GBV intervention in particular were affected considerably by contact restrictions owed to the Covid-19 pandemic [27, 28] (see also CONSERVE checklist [29] in appendix).

### Randomization

Random allocation sequence to the control (TAU) or intervention (GBV plus TAU) group with a 1:1 allocation was created independently by the Institute for Epidemiology and Medical Biometry at Ulm University using the ROM software according to a computer-generated randomization schedule stratified by site using permuted blocks of random sizes (small sites: 2, 4 or 6; medium sites: 4, 6 or 8; big sites: 8, 10, or 12). Study participants were informed about the outcome of the randomization by study workers after the baseline assessment was completed.

### Study measures

In line with national [30] and international [13] treatment guidelines for the target group of people with SMI, the improvement of empowerment, i.e. a person’s perceived control over her or his life was chosen as primary outcome. The Assessment of Empowerment in Patients with Affective and Schizophrenic Disorders (EPAS) is a self-evaluation questionnaire of 33 items measuring empowerment as the patient’s perceived control of his or her life on five dimensions: daily living, social relationships and sexuality, psychiatric treatment, hope and self-efficacy and self-esteem [31].

Secondary outcome measures comprised the following questionnaires:

- The Health of the Nation Outcome Scale (HoNOS) is an expert assessment instrument measuring the clinical and psychosocial impairment due to a mental disorder on twelve dimensions, independent of diagnosis [25, 32].
- The Camberwell Assessment of Need (CAN) is a self-rating instrument for the assessment of mental health service needs and the extent to which these needs are met or not by available services covering 23 areas [26, 33].
- The service satisfaction questionnaire (ZUF-8) [34] is the German short version of the Client Satisfaction Questionnaire (CSQ-8) [35], a self-rating questionnaire including 8 items measuring patient satisfaction with their mental health care.
- The WHO Quality of Life (WHOQOL-BREF) instrument [36–38] is a self-rating questionnaire including 25 items measuring patients’ subjective quality of life on the dimensions of physical health, mental well-being, social relationships, and environmental conditions.

### Harm outcomes

We defined serious adverse events (SAE) as all events resulting in the death, intended self-injury, or a life-threatening situation of a study participant. The participants were asked to immediately report SAEs to the study workers or to GBV staff (if they were in the GBV group). All research workers explicitly asked for SAEs during follow-up assessments.

### Assessment procedure

Assessments for study eligibility were conducted by clinical staff members of community mental health service providers trained by members of the research team [18].

Assessments in the evaluative trial were conducted by research workers trained by members of the research team. Due to the restrictions of personal contact during the Covid-19 pandemic, assessment was conducted either face to face or by video call depending on the pandemic situation and the preferences of the study participants as individual sessions with research workers, independent of health care provision. During the course of the study remote contacts with the study team became the preferred option for the majority of participants (t0: 45%, t4: 87%). Self-rating questionnaires (EPAS, ZUF-8 and WHOQOL-BREF) were answered by participants alone at electronic devices. In case of problems with internet access data were collected by paper-pencil and entered into the data entry system by research workers.

### Blinding

Blinding of research workers was not possible because the detailed assessment of service utilization including the services of the GBV intervention was part of the data collection.

### Statistical methods

Sample size estimation was based on a linear mixed effects regression model with five points of measurement (t_0_ – t_4_) at twelve study sites using the RMASS software [39]. Based on the results of a previous study [17] we expected an effect size of the primary outcome parameter increasing from 0.0 standard deviations (sd) at t_0_ to 0.2 sd at t_4_ and a total study dropout rate of 28% from t_0_ to t_4_. Using a power of 0.9 at a significance level of *p* ≤ 0.05 we estimated an initial sample size of *n* = 978.

Linear mixed effects regression (LMER) models for panel data including linear parameters for time, treatment and time*treatment interactions were applied to estimate outcome effects. LMER using full information maximum likelihood estimators (FIML) allows the inclusion of the total sample by taking into account missing observations under the missing at random (MAR) assumption [40].

Difference in difference (DiD) effect sizes were estimated as Cohen’s d for the difference in the change between t_0_ and t_4_ between the TAU and the GBV group on an intention to treat (ITT) basis. Multiple imputation of missing values (with 20 imputations) was conducted using the regression method with gender, age, ICD-10 diagnosis, health insurance provider, having a job, living in sheltered accommodation and study site at baseline as independent variables. Analyses were conducted according to the combination rules suggested by Rubin [41].

Mixed effects regression models were conducted with the mixed procedure, effect sizes were computed by the esize module provided by STATA 17 [42].

Differences in the number of reported SAEs were tested by means of the Pearson Chi^2^ test or by the Fisher exact test using the t_0_ sample sizes as denominator.

## Results

**Fig. 1:**
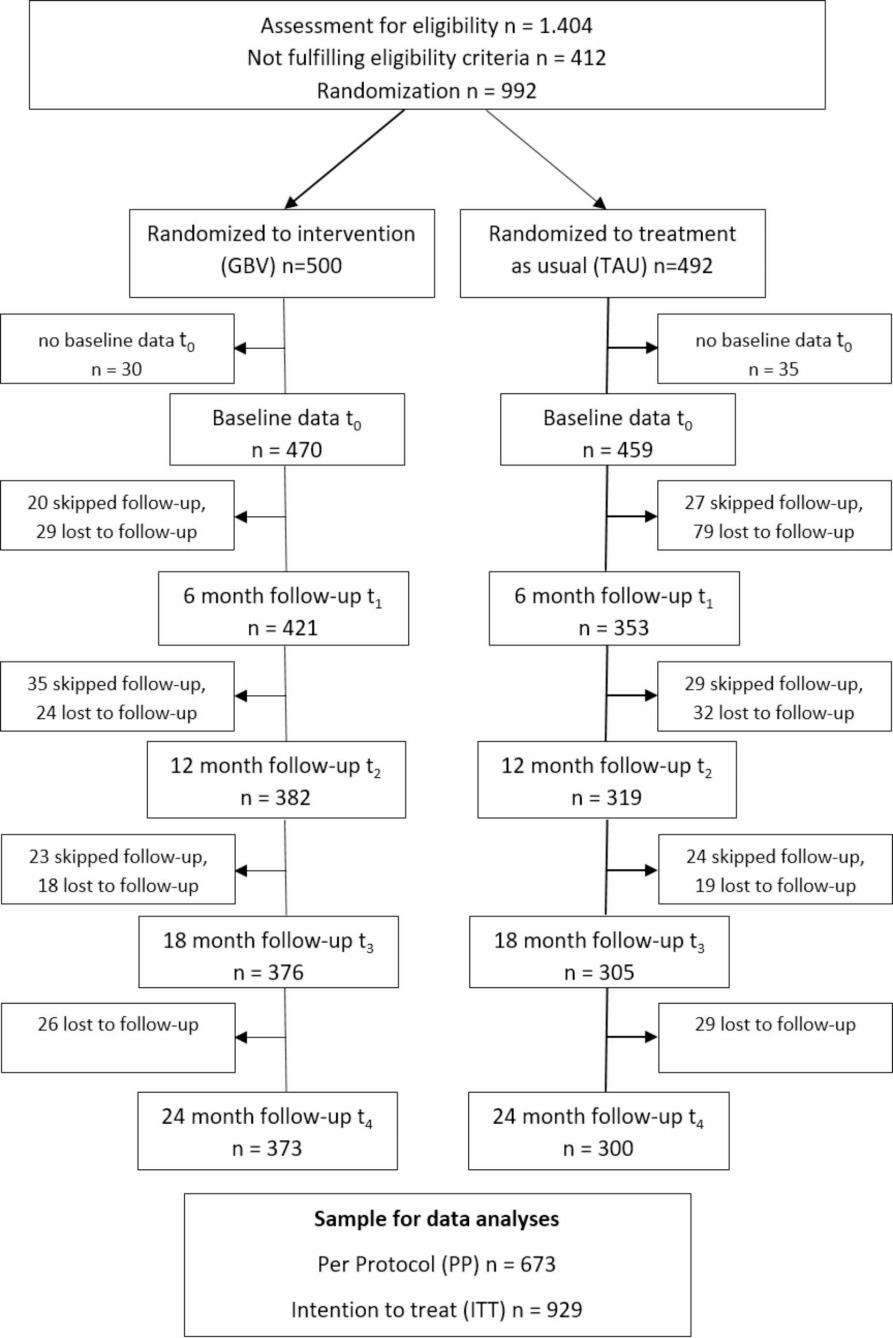
Study flowchart

As indicated in Fig. 1, out of 1,404 persons screened 992 were found to be eligible for study participation and randomly assigned to the two study groups. Due to withdrawal of consent, 63 persons were excluded from further participation and data analysis. A total of 929 participants (TAU, *n* = 459 and GBV, *n* = 470) were included in the ITT analyses. Due to the loss of 256 participants (TAU, *n* = 159 and GBV, *n* = 97) to follow-up assessment the per-protocol sample was *n* = 673.

A total of 32 serious adverse events (SAE) were reported. Four events resulted in deaths, three in the GBV and one in the TAU group. In the TAU group life-threatening events were reported by 16 participants (3.5%), and in the GBV group 12 participants (2.6%) reported life-threatening events. Apart from the four participants who deceased during the follow-up period, no further participants dropped out of the study due to SAE.

As indicated in Table 1, about 63% (*n* = 582) of the participants were female with a mean age of 42 years (sd = 13.2 yrs.). A majority of 56% (*n* = 520) of the participants had a higher school education (Abitur and above), and 92% (*n* = 856) were German citizens. While 36% (*n* = 334) reported having a partner, 45.5% (*n* = 421) lived alone and 41.8% (*n* = 388) indicated to be employed. A proportion of 80% (*n* = 744) were health insured with the Techniker Krankenkasse (TK) which is the biggest statutory health insurance provider in Germany. Regarding clinical characteristics, participants indicated an average duration of mental illness of 15 years (sd = 13 years) and an average number of psychiatric inpatient admissions of 2.4 (sd = 4.0). The majority of participants had a diagnosis of an affective disorder (*n* = 629; 67.7%) followed by anxiety disorders (*n* = 136; 16.8%), schizophrenia spectrum disorder (*n* = 85; 9.3%), and other psychiatric diagnoses (*n* = 59; 6.2%). Results of the Chi^2^ test or the t-test revealed no significant differences between sample characteristics or the outcome measures at baseline, neither for both study groups nor for study completers and dropouts.

Participants in the intervention group reported up to 69 contacts with GBV staff per quarter, with decreasing number of contacts over time (t1: median = 5, t2 + t3: median = 4, t4: median = 3) and a median of four contacts per quarter over 24 months of intervention.

**Table 1:** Sociodemographic, clinical characteristics and outcome at baseline

	Total *n* = 929	TAU *n* = 459	GBV *n* = 470	*p* diff^1^
**Sociodemographic characteristics**				
Female n (%)	582 (62.8)	288 (63.0)	294 (62.6)	0.136
Age m (SD)	42.4 (13.2)	42.3 (13.3)	42.5 (13.1)	0.807
Higher education n (%)	520 (56.1)	252 (55.1)	268 (57.0)	0.564
Having a partner n (%)	334 (36.0)	161 (35.2)	173 (36.8)	0.617
Living alone n (%)	421 (45.5)	206 (45.1)	215 (45.8)	0.815
Employed n (%)	388 (41.8)	201 (43.9)	187 (39.8)	0.206
German citizen n (%)	856 (92.3)	428 (93.7)	428 (91.1)	0.138
Monthly income above 2,500 € n (%)	308 (34.5)	154 (35.2)	154 (33.8)	0.662
Health insurance TK n (%)	744 (80.3)	358 (78.3)	386 (82.1)	0.633
**Clinical characteristics**				
Years mentally ill m (SD)	15.1 (13.0)	14.9 (12.5)	15.4 (13.4)	0.697
Inpatient admissions m (SD)	2.4 (4.0)	2.4 (3.8)	2.4 (4.3)	0.430
Psychiatric diagnosis group ICD-10				
-Psychosis (F2) n (%)	85 (9.2)	46 (10.1)	39 (8.3)	0.362
-Affective (F3) n (%)	629 (67.7)	307 (66.9)	322 (68.5)	0.596
-Anxiety (F4) n (%)	156 (16.8)	77 (16.8)	79 (16.8)	0.989
-Other n (%)	59 (6.4)	29 (6.3)	30 (6.4)	0.968
**Baseline outcome measures**				
EPAS total score m (SD)	2.97 (0.54)	2.98 (0.53)	2.96 (0.55)	0.549
HoNOS total score m (SD)	15.27 (	15.26 (5.53)	15.28 (5.35)	0.959
WHOQOL-BREF m (SD)	37.27 (20.26)	37.25 (20.02)	37.29 (20.51)	0.980
ZUF-8 m (SD)	20.72 (4.97)	20.47 (4.99)	20.96 (4.93)	0.139
CAN number of unmet needs / number of total needs m (SD)	0.69 (0.27)	0.70 (0.27)	0.68 (0.27)	0.191

^1^ Pearson’s Chi^2^ tests were conducted for categorical variables and Students t-tests were applied with continuous variables

TAU = Treatment as usual (control group); GBV = Gemeindepsychiatrische Basisversorgung (intervention group); EPAS = Assessment of Empowerment in Patients with Affective and Schizophrenic Disorders; HoNOS = Health of the Nations outcome Scale; WHOQOL-BREF = WHO Quality of Life Questionnaire Brief Version; ZUF-8 = Service Satisfaction Questionnaire; CAN = Camberwell Assessment of Needs

**Fig. 2:**
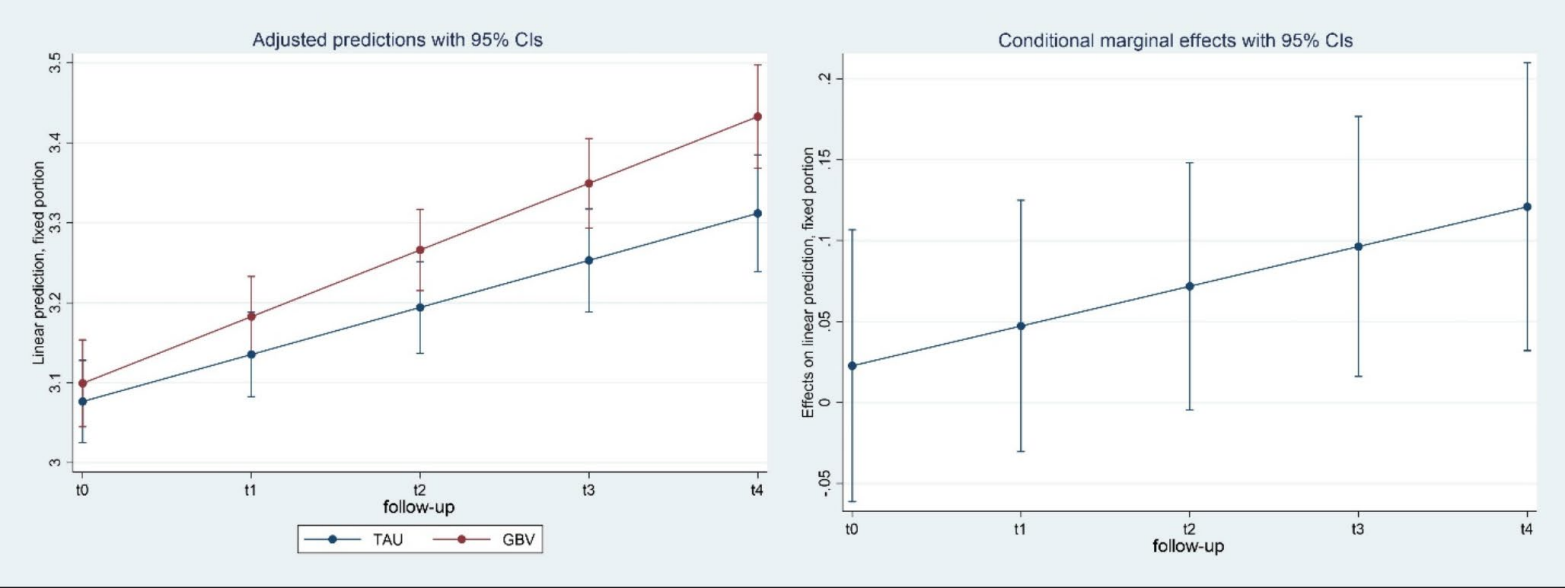
Marginal means and marginal effects GBV vs. TAU in EPAS total score

Results of the mixed effects regression models are presented in Table 2. The regression coefficient for the time effect in the primary outcome measure EPAS total score (b = 0.07; p < = 0.001) indicates that the perceived level of empowerment in the reference group TAU significantly increased by 0.07 units at each follow-up. The regression coefficient for the time*treatment interaction effect (b = 0.03; p < = 0.001) indicates that the improvement of empowerment at each follow-up was significantly larger in the GBV group (by 0.03 units) compared to the TAU group. Marginal effects of the linear prediction for the primary outcome EPAS total score are presented in Fig. 2. The total DiD effect size was d = 0.27 (95% CI = 0.14 0.40).

**Table 2:** Results of the mixed effects regression model for the primary outcome and the secondary outcomes

	*N*	Time b (95% CI)^1^	GBV vs. TAU b (95% CI)^1^	Time * GBV vs. TAU b (95% CI)^1^	Constant b (95% CI)^1^	Cohen’s d t4 – t0 (95% CI)
EPAS total	923	0·07*** (0·05 0·08)	0·00 (-0·09 0·09)	0·03*** (0·01 0·05)	2·99 (2·96 3·04)	0.27 (0.14 0.40)
HoNOS total score	927	-1·24*** (-1·62 -0·86)	-0·31 (-0·75 0·13)	0·12 (-0·04 0·28)	14·8 (14·08 15·54)	0.07 (-0.14 0.16)
WHOQOL-BREF	922	2·37*** (1·83 2·91)	0·59 (-1·30 2·49)	0·87** (0·36 1·38)	38·77 (36·91 40·63)	0.22 (0.09 0.35)
ZUF-8	922	0·44*** (0·31 0·57)	1·34 (0·76 1·93)	0·40*** (0·23 0·58)	20·52 (19·97 21·07)	0.41 (0.28 0.54)
CAN Number of unmet needs / total number of needs	927	-0·04*** (-0·05 -0·04)	-0·53** (-0·08 -0·02)	-0·02*** (-0·02 − 0·01)	0·68 (0·60 0·75)	-0.26 (-0.39 -0.13)

^1^ Robust standard errors to take into account clustered variance at 12 sites

EPAS = Assessment of Empowerment in Patients with Affective and Schizophrenic Disorders; HoNOS = Health of the Nations Outcome Scale; WHOQOL-BREF = WHO Quality of Life questionnaire Brief version; ZUF-8 = Service Satisfaction Questionnaire; CAN = Camberwell Assessment of Needs

LMER for the secondary outcomes indicated significant time*treatment effects also for the increase of quality of life and service satisfaction and for the decrease of the share of service need which was not met by the services received. No significant treatment effect was found for clinical and functional disabilities related to mental disorder (see Table 2):

The regression coefficient for the HoNOS total score (b = -1.24; p < = 0.001) reveals that the level of disease-related disability, in participants assigned to the TAU group, decreased significantly by 1.24 at each follow-up. The non-significant coefficient for the time*treatment interaction effects indicates that there was no significant difference in the change of disability levels between the study groups. This was also confirmed by an effect size of d = 0.07 (95% CI = -0.14 0.16).

Regression coefficients for the WHOQOL-BREF overall score indicate a significant improvement of quality of life by 2.37 units at each follow-up in the TAU group (b = 2.37; p < = 0.001) and a significant 0.87 units larger increase (b = 0.87; p < = 0.01) per follow-up in the GBV group compared to the TAU group. The DiD effects size for the WHOQOL-BREF was d = 0.22 (95% CI = 0.09 0.35).

The regression coefficients for the ZUF-8 score indicate that participants’ satisfaction with mental health services increased by 0.44 units at each follow-up in the TAU group (b = 0.44; p < = 0.001) and that the increase was by 0.4 units per follow-up higher (b = 0.40; p < = 0.001) in the GBV group compared to the TAU group. The DiD effects size for the ZUF-8 was d = 0.41 (95% CI = 0.28 0.54).

Regression coefficients for the proportion of unmet needs to total needs indicate a significant linear decrease in the TAU group (b = -0.04; p < = 0.001) and a stronger decrease in the GBV group compared to TAU (b = -0.02; p < = 0.001). The DiD effects size for the proportion of unmet needs was d = 0.26 (95% CI  = 0.13 0.47).

**Fig. 3:**
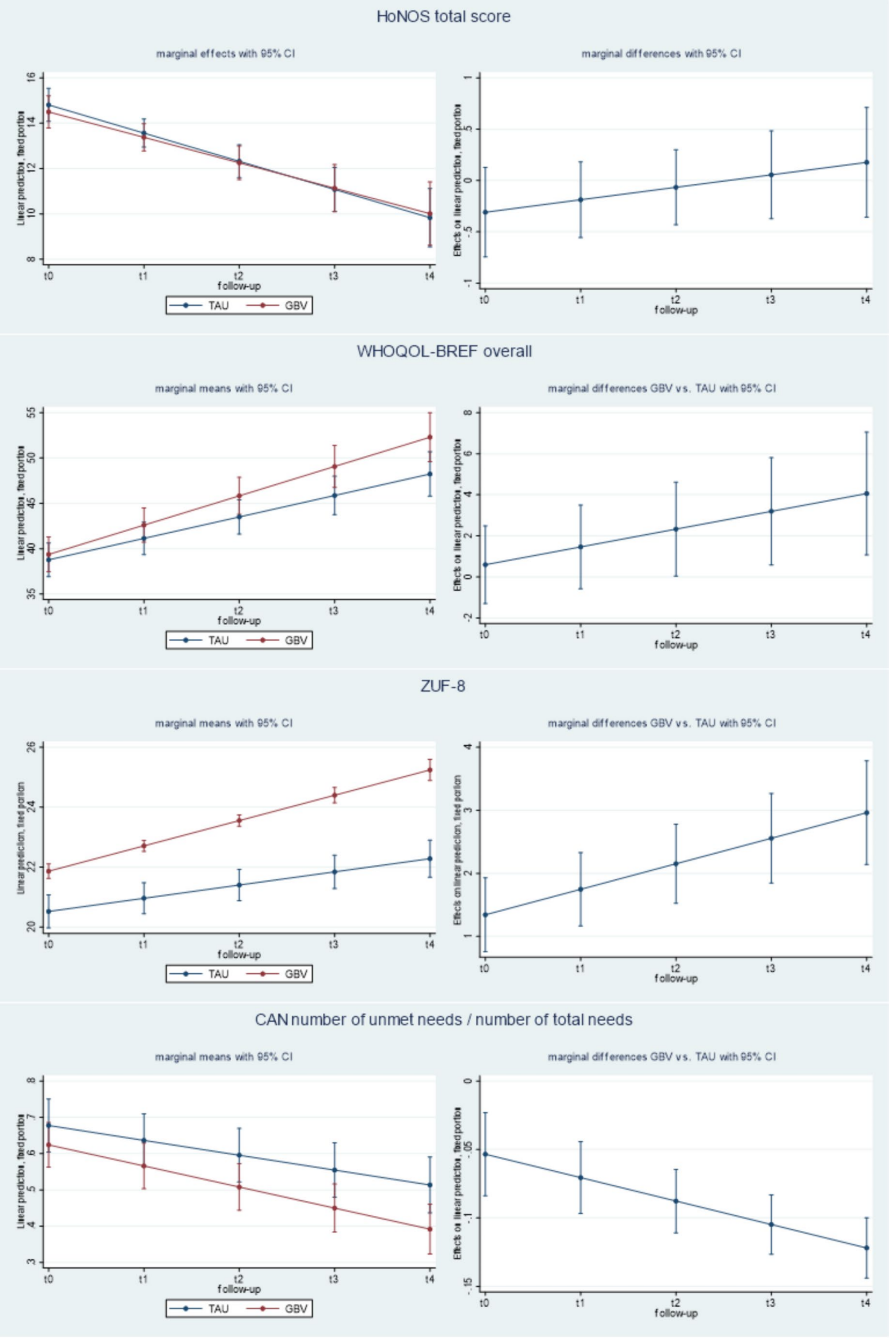
Marginal means and marginal differences GBV vs. TAU in HoNOS total score, WHOQOL-BREF overall score, ZUF-8 total score and CAN proportion of unmet needs to total needs

Marginal effects of the secondary outcome measures are presented in Fig. 3.

All findings were replicated in the multiple imputed data sets (see Table 3).

**Table 3:** Results of the mixed effects regression models for primary and secondary outcomes with multiple imputed data

	Time b (95% CI)^1^	GBV vs. TAU b (95% CI)^1^	Time * GBV vs. TAU b (95% CI)^1^	Constant b (95% CI)^1^	Cohen’s d t4 – t0 (95% CI)
EPAS total	0.59*** (0.05 0.087)	-0.003 (-0.09 0.09)	0.02** (0.0 0.04)	3.02 (2.97 3.07)	0.27 (0.14 0.40)
HoNOS total score	-0.97*** (-1.26 -0.68)	-0.21 (-0.68 0.21)	0.03 (-0.16 0.22)	14.8 (13.82 15.23)	0.07 (-0.14 0.16)
WHOQOL-BREF	2.37*** (1.83 2.91)	0.59 (-1.30 2.49)	0.87** (0.36 1.38)	38.77 (36.91 40.63)	0.22 (0.09 0.35)
ZUF-8	0.44*** (0.31 0.57)	1.34 (0.76 1.93)	0.40*** (0.23 0.58)	20.52 (19.97 21.07)	0.41 (0.28 0.54)
CAN Number of unmet needs / total number of needs	-0.04*** (-0.05 -0.04)	-0.53** (-0.08 -0.02)	-0.02*** (-0.02 -0.01)	0.68 (0.60 0.75)	0.26 (0.13 0.47)

^1^ Robust standard errors to take into account clustered variance at 12 sites

EPAS = Assessment of Empowerment in Patients with Affective and Schizophrenic Disorders; HoNOS = Health of the Nations Outcome Scale; WHOQOL-BREF = WHO Quality of Life questionnaire Brief version; ZUF-8 = Service Satisfaction Questionnaire; CAN = Camberwell Assessment of Needs

## Discussion

This multi-center RCT, with twelve study centers spread nationally, is the first trial investigating the effectiveness of a community-based mental health (GBV) intervention on the level of perceived empowerment among people with SMI in Germany. Its results indicate that participants who received the GBV intervention, in addition to TAU, over a 24-month period reported a larger improvement of their level of empowerment than did those study participants who received TAU alone. Although the effect size for the overall empowerment score as primary outcome of this study was low, the continuous increase over the consecutive time points of study follow-up indicates a sustained improvement of empowerment among people with SMI in what looks like a slow and steady process coming into effect over time. We found no differential intervention effects on the severity of disability related to mental illness measured by the HoNOS total score. This suggests that the GBV intervention appears not to cause any effects on patients’ clinical characteristics such as psychopathological symptoms, behavioral problems, or social role functioning when compared to the TAU treatment condition. Participants in the GBV group improved significantly more in subjective quality of life and in their satisfaction with mental health services when compared to participants who received TAU alone. The effects on service needs suggest that the GBV intervention significantly improved the fit between mental health service provision and clients’ needs among people with SMI.

In Germany, there is one other study, an observational controlled trial, comparing the effects of an integrated community mental health care program with standard care for people with SMI using empowerment as the primary outcome. After 18 months the authors found no treatment effects on primary or secondary outcomes [17, 43]. Potential reasons for the lack of effectiveness were the preference based group allocation, the sampling procedure applied in this study and the per capita lump sum payment for the services [17, 43].

Comparing our results with respect to user empowerment in persons with SMI with those of further studies is difficult because empowerment has rarely been used as a primary outcome and studies using it have applied different measures and varied in their definitions of target groups and type of intervention [7, 44–50]. Swildens and colleagues [47] used empowerment as a secondary outcome in a study on the effectiveness of the Boston Psychiatric Rehabilitation Approach but found no treatment effect on this parameter. Tjaden and colleagues [7] investigated the effects of combining flexible assertive community treatment (FACT) with the resource group (RG) method and found an effect size of d = 0.54 with respect to empowerment. Porter & Bejerholm [49] evaluated the effectiveness of an individual enabling and support program compared to a traditional vocational rehabilitation program on the empowerment of patients with depression over twelve months and found a small effect size with regard to the improvement of empowerment. In a study on the effectiveness of an intervention for the rehabilitation and self-management among patients with treatment-resistant depression or anxiety disorder, Zoun and colleagues [51] found an effect size of d = 0.45 for the improvement of empowerment. In an evaluation of the effectiveness of a self-management program for people with SMI during 24 months following hospital discharge Lay and colleagues [50] found no significant treatment effects on the improvement of empowerment. As a result of a meta-analysis on the effectiveness of peer-delivered recovery education interventions Peck and colleagues [44] found a mean effects size of d = 0.46 on empowerment-related outcomes. Compared to these studies the effects revealed in our investigation were considerably smaller. However, since personal communication and networking activities are crucial elements of the GBV intervention [18] we assume that the restrictions of personal contact owed to the Covid-19 pandemic could have had an impact on effect sizes.

With regard to the secondary outcome measures, the findings of our study are partly in accordance with the results of a recent Cochrane review on the effectiveness of intensive case management (ICM) compared to standard care [14, 15] indicating no overall effects of ICM on general symptoms, social functioning, or behavioral outcomes but a significant effect on patient satisfaction with mental health care. Dieterich et al. did not look for effects of ICM on empowerment, and they identified only one study indicating an effect of ICM on QOL [14, 15]. Taken together, this indicates that ICM in general, but also GBV specifically, promotes personal recovery, but not clinical recovery. This is not surprising given that personal and clinical recovery are based on different understandings of recovery and that that clinical measures fail to assess important aspects of consumer-defined recovery [52], which the GBV aimed to address.

### Strengths and limitations

The strengths of this study include the RCT design, the inclusion of participants from different study sites across Germany, the large sample size in combination with a low panel attrition rate, and a study duration of 24 months.

Another strength is the approach for patient and public involvement as applied in this project. The overall project and the implementation of the intervention was led by the German umbrella association for community psychiatry (Dachverband Gemeindepsychiatrie) which represents professional providers from a wide variety of legal forms as well as social space-oriented support services provided by citizens and self-help organizations for people living with mental illness and their relatives. The consortium, especially one coordinator with lived experience (EP), made sure that service users’ views were sought and taken into account during implementation of the GBV intervention and during evaluation of its effectiveness. Moreover, as part of process evaluation, qualitative interviews with service users were conducted and will provide further insights on the service users’ experiences with the GBV and subjective implications of the GBV intervention on health care provision, also related to empowerment.

A major limitation results from the fact that the participants and study workers who conducted the data collection were not blinded with regard to the intervention. A limitation of the generalizability of the study outcomes may result from the fact that 80% of study participants were insured with one single health insurance company out of about 95 statutory health insurance providers. A further limitation results from having conducted the study under strong restrictions of face-to-face communication due to the Covid-19 pandemic that could have contributed to the small intervention effects. It might also be questioned if the small effects detected are clinically important.

In view of the chosen outcomes, it must be admitted that further outcomes of interest were neglected in this study: Thus, we had only focused on the personal dimension of empowerment and neglected the social (e.g. social inclusion) and political (e.g. access to rights and services) dimension of this concept in the effectiveness evaluation. As these dimensions were intended to be addressed by GBV teams and are also covered in the quality standards of the intervention, it is possible that some benefits of the GBV could not be revealed as part of this RCT. However, we are exploring these topics as part of the above mentioned qualitative interview study with 15 participants from the intervention group. From a holistic point of view, another limitation of this trial in patients with SMI was that physical health outcomes were not addressed, neither by the intervention itself nor as study outcomes. In line with pivotal clinical guidelines such as the Blueprint for Protecting Physical Health [53], the concept should be expanded with regard to the integration of physical health care and lifestyle interventions.

## Conclusion

Results of this study indicate that, even under the adverse conditions of the Covid-19 pandemic, the implementation of the GBV intervention in addition to routine mental health care in Germany was associated with a stronger improvement of empowerment when compared to treatment as usual alone. Thus, the implementation of such an intervention can be considered an effective measure to improve key psychosocial outcomes among people with severe mental illness.

## Data Availability

The data that support the findings of this study are available from the corresponding author, [AMS], upon request.

## Analytical code availability

The analytical code is available from the corresponding author, [AMS], upon request.

## Research material availability

Material related to the paper (in German) will be available on the website of the funding agency (https://innovationsfonds.g-ba.de/projekte/neue-versorgungsformen/gbv-gemeindepsychiatrische-basisversorgung-schwerer-psychischer-erkrankungen.280).
